# Aconite in Fever

**Published:** 1894-10

**Authors:** C. Carleton Smith

**Affiliations:** Philadelphia, Pa.


### ACONITE IN FEVER.

Editor of The Homœopathic Physician:—The terse and pointed remarks made upon Aconitum-nap. in your editorial in September number of The Homœopathic Physician are worthy of constant reiteration.

This drug, though one of the most precious in our materia medica, is not by any means a specific for fever, as many authors and practitioners would have us believe. Persons studying this remedy from the standpoint of the late Dr. Hempel, as we find it set forth in his voluminous *Materia Medica*, and not being acquainted with the genius of the homœopathic law, would at once conclude that Aconite is not only a specific for fever, so-called, but that it is a specifie remedy as well for all the ills that mankind is heir to. The fact is, there is no such thing as a specific in medicine. None has ever been discovered, and none ever will be. Why? For the simple reason that the sick individual must be treated, and not the disease from which he is sufferiug. And as no two individuals present precisely the same group of symptoms though ill from the same disease according to its nomenclature, it stands to reason that there never can be such a thing as a specific in medicine.

To state the truth, Aconite does not cure fever. If it does, then almost every drug in the materia medica has the same power. But it does cure *a fever*, whenever it is given the opportunity to do so, and that fever is and always will be the fever which it alone is capable of producing in its proving upon the healthy individual. That, and only that. Nothing more, nothing less. Used in this way, strictly in accordance with its symptomology, as all homœopathically proven drugs should be used, its curative action will be as sure as the daily rising and setting of the sun, and ofttimes it will border on the miraculous.

This business of treating a fever, which as a symptom is almost always present in a greater or lesser degree in nearly every case of illness, with the sole purpose in view of reducing the high temperature, by Aconite or any other drug, is not only absurd, but it is a display of ignorance that should have no place in homœopathic ranks. It is a fatal error to endeavor to reduce or abort the fever in any case of eruptive disease, by prescribing for the fever alone, as though that one single symptom were the all in all. Just here is where the drug in question has been abused time out of mind, to the peril of the patient, its exhibition in some cases being followed by death. Let it be understood by such prescribers that the excess of heat, commonly called fever, is nature's method of throwing the eruption to the surface. Therefore it should not be tampered with. Keep your Aconite out of sight unless it covers completely all the characteristic symptoms of the case in hand; in which event the patient as well as the fever will be cured, and that homœopathically, by this particular drug. Do not let us be guilty of straddling certain remedies and riding them to death as specifics. Whenever I hear a doctor talking about giving Aconite for fever, Nux-vom. for piles, Sepia for prolapsus-uteri, and Bryonia for rheumatism, etc., etc., I am constrained to think that the sooner he takes down his sign, the better it will be for himself and for the public.

C. CARLETON SMITH.

PHILADELPHIA, PA.

